# Characteristics of cavitation onset and development in a self-excited fluidic oscillator

**DOI:** 10.1016/j.ultsonch.2022.106018

**Published:** 2022-04-29

**Authors:** Gang Liu, Haiyan Bie, Zongrui Hao, Yue Wang, Wanlong Ren, Zhili Hua

**Affiliations:** a Institute of Oceanographic Instrumentation, Qilu University of Technology (Shandong Academy of Sciences), Qingdao 266100, China; b College of Chemistry and Chemical Engineering, Ocean University of China, Qingdao 266100, China

**Keywords:** Hydrodynamic cavitation, Gray intensity, Cavitation number, Visual experiment

## Abstract

Hydrodynamic cavitation has been widely employed in modern chemical technology. A high-speed camera experiment is conducted to reveal the characteristics of hydrodynamic cavitation generated in one self-excited fluidic oscillator. The images obtained from the high-speed camera system are employed to describe several development stages of the hydrodynamic cavitation. The gray intensity of the images which is the volume of bubbles formed is extracted to distinguish the cavitation bubbles from the water. It is found that three regions in the fluidic oscillator could be divided according to the distance from the entrance. The inception of cavitation occurs in the region nearest the entrance. For a relatively low inlet flow rate, the whole process of cavitation could complete within the region that is the second nearest the entrance as a low pressure area appears periodically in this region. For a high inlet flow rate, the vortexes in the region farthest from the entrance are able to generate sufficient low pressures to induce the generation of cavitation. In addition, the intensity of cavitation could be reflected by the cavitation number in a self-excited fluidic oscillator.

## Highlights

• Characteristics of cavitation in a fluidic oscillator is explored experimentally.
• The gray intensity of the images is extracted to distinguish the cavitation bubbles.
• Three regions are chosen to describe the details of cavitation.
• Intensity of cavitation is reflected by the cavitation number in a fluidic oscillator.

## 1 Introduction

As a kind of complex flow phenomenon, hydrodynamic cavitation has always been attracting extensive attention from scholars [1], [2], [3]. Numerous negative effects including material erosion, noise and vibration on the mechanical equipment may be induced by the hydrodynamic cavitation [4], [5], [6]. Nevertheless, due to the tremendous power that could be generated during the development of hydrodynamic cavitation, a variety of cavitation reactors have been created and then applied in various physical and chemical processes such as hydrolysis of oils and water treatment [7], [8], [9], [10]. Particularly, the microbubbles which have great advantages in chemical reactions [11] could be generated in the process of cavitation [12]. The ammoniacal nitrogen in wastewaters could be removed by the enormous amount of energy released during cavitation bubbles collapse [13], [14].

In order to make full use of the energy of cavitation, several common kinds of cavitation reactors including the Venturi and Orifice type have been employed in the last decade as water could be forced to pass through a constriction device to generate cavitation in these reactors [15], [16]. It seems that the Venturi reactors have always been explored by scholars [17], [18], [19], [20], [21]. Due to the existence of the throat part, the water injected into the Venturi reactor suffers a stage that the pressure sudden increasing and then sudden decreasing. The sharp reduction in pressure would prompt the generation and development of hydrodynamic cavitation [20]. Brinkhorst et al. [18] presented that periodic cavitation shedding could be observed in the Venturi reactor. It was the periodic fluctuations of pressure generated in the Venturi reactor that induce the hydrodynamic cavitation. As the fluctuations of pressure is only formed due to the particular structure of the Venturi reactor, this kind of simple reactor has been widely employed to create hydrodynamic cavitation.

Actually, pressure fluctuations could also exist in some other reactors such as the self-excited fluidic oscillator. This fluidic oscillator is also a kind of reactor with simple structure which has been widely employed in flow separation control [22], drag reduction [23], heat transfer enhancing [24] and so on. A steady state jet could be converted into an oscillatory one according to the intrinsic flow instability in a fluidic oscillator. In a Venturi reactor, the liquid would suffer a lower pressure than the saturated vapor in the throat section with a narrowing diameter. Cavitation then may occur at this region. While in one self-excited fluidic oscillator, a stable main jet would oscillate continuously in the chamber without any moving parts. During this process, violent fluctuations also occur for the pressures, which may generate intense cavitation. It should be noted that the fluctuations of pressure in the chamber of the fluidic oscillator is mainly driven by the oscillation of the main jet [25], [26], which depends on the effects of feedback channels. Liu et al. [27] suggested that large bubbles could be broken into microbubbles under the actions of the oscillating flow field. Then it is speculated that the oscillating flow field in the chamber may be able to prompt the process of hydrodynamic cavitation. Whether cavitation can occur in the fluidic oscillator would be issued in this work.

The phenomenon of hydrodynamic cavitation has been researched by numerical simulations frequently in the past decade [18], [19], [28], [29], [30], [31]. Although the numerical simulations can easily obtain the flow field information of cavitation process, the experimental data may be more reliable to understand the essence of the cavitation. Because a large number of transient cavitation bubbles occur during this process, high-speed photography maybe the best experimental method.

The bubble breakup in the Venturi reactor was explored by the high-speed camera technology in the work of Wang et al. [11] Song et al. [32] and Huang et al. [33]. For cavitation phenomenon, the dynamics of the cavitation bubbles generated in certain holes of PMMA plates were explored via a high-speed camera system in the work of Kauer et al. [34]. The high-speed visualization technology was employed to investigate a kind of vortical cavitation by Karathanassis et al. [35]. Guo et al. [36] explored the cavitation inside the multi-orifice plates with the help of PIV system and high-speed camera system. Li et al. [37] conducted a high-speed camera experiment to discuss effects of air bubbles on the cavitation development. Bai et al. [38] focused on the deformation process of cavitation bubbles. Nevertheless, it seems that the overall development characteristics of cavitation clouds inside one fluidic oscillator have not been explored in depth, which is the main object of this work. Since there are great differences between the pixels for the bubbles and water, in the work of Liu et al. [39], the gray intensity of the photographs obtained by the high speed camera was discussed to reveal the process of cavitation.

In order to explore the cavitation phenomenon in the fluidic oscillator, a high-speed camera experiment is conducted in this work. The images obtained from the high-speed camera system are used to describe the developmetn of the hydrodynamic cavitation. The gray intensity of the images is extracted to distinguish the cavitation bubbles from the water. The characteristics of cavitation under three inlet flow rates are discussed.

## 2 Experimental setup

**Fig. 1:**
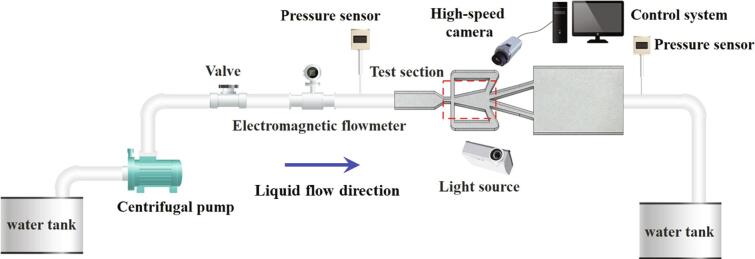
Sketch of the experimental system.

The fluidic oscillator cavitation system is depicted in Fig. 1. A centrifugal pump is employed to supply water (25 °C) with sufficient flow rate, which is measured by an electromagnetic flowmeter. It is noted that 20.5 mg air dissolved in every liter of water. The pressures at the inlet and outlet regions are monitored by two high-frequency pressure sensors. The liquid flow rates were mainly adjusted by the frequency converter matched with the water pump. Nevertheless, in order to further controlling the liquid flow rates, one valve was installed in the experimental loop. In the experiment for any certain flow rate, the opening of the valve remains unchanged all the time.

**Fig. 2:**
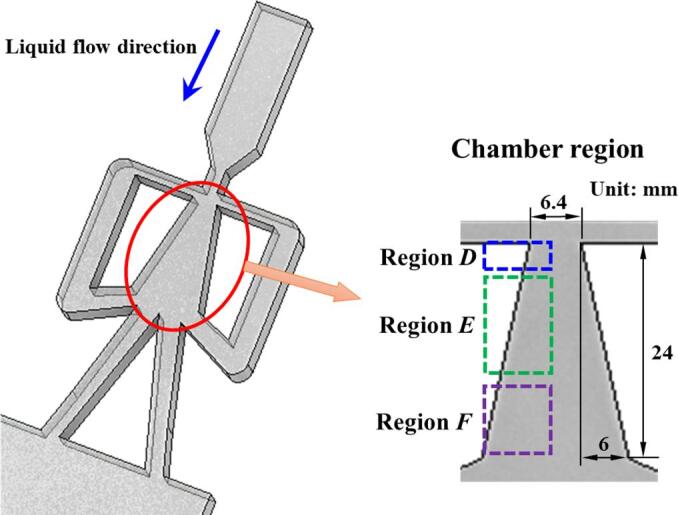
Schematic diagram of the self-excited fluidic oscillator.

The chamber region of the fluidic oscillator as depicted in Fig. 2 is the main test section. The liquid flow direction and the sizes of the chamber could be seen in Fig. 2. The behaviors of cavitation in the chamber are recorded by the high-speed camera system. As the flow fields perform diverse characteristics as the movement of the main jet, three sub-regions including Region *D*, Region *E* and Region *F* are compartmentalized to discuss the details of cavitation. The test section is photographed through a high-speed camera system by the backlight source method. In order to capture the details of cavitation and obtain a preferable shooting effect, the shooting frame is set as 3600 fps.

As noted in the work of Wu et al. [25], Hao et al. [26] and Liu et al. [27], the oscillations of the main jet mainly occur in the chamber region of the fluidic oscillator. Then only this region would be concerned in the high-speed camera experiment.

The development of the cavitation process under three cases is discussed firstly. These discussions could be obtained directly from the images. The variations of the cavitation which may induced by the oscillations of the main jet are explored in detail, which could reveal the characteristics of the cavitation preliminarily.

The image processing method is employed to analyze the unsteady characteristics of the transient cavitation flow. The image for cavitation could be obtained by the high-speed camera system. Under the irradiation of light, the reflectance of the incident light is different between liquid and cavitation bubble. Then there are significant differences in luminance between water and cavitation bubble regions. Therefore, the distributions of gray-level of the photographs could be detected to distinguish the region of the cavitation bubbles and the water. The following matrix mentioned in the work of Liu et al. [39] is introduced to analyze each cavitation image:

(1) $$Image\left(n\right)=\left[\begin{array}{ccc} A\left(1,1,n\right) & \cdots & A\left(1,j,n\right)\\ \vdots & \ddots & \vdots \\ A\left(i,1,n\right) & \cdots & A\left(i,j,n\right) \end{array}\right]$$

where *A* represents the grayscale and *n* denotes the referenced number for images. *i* and *j* stand for the row and column of the image matrix severally. It should be noted that for the grayscale *A* (*i*, *j*, *n*)∈{0, 1, …,255} (0 for black pixel and 225 for while pixel).

The operating conditions in the experiments are shown in Table 1. Three different flow rates of liquid are selected to explore the variations of cavitation clouds.

**Table 1:** Operating conditions in the experiments.

		*p*_1_ (MPa)	*p*_2_ (MPa)
*Q_in, L_* = 1.745 m^3^/h	*Re_in, L_* = 44065	0.098	0.005
*Q_in, L_* = 2.082 m^3^/h	*Re_in, L_* = 52576	0.144	0.005
*Q_in, L_* = 2.496 m^3^/h	*Re_in, L_* = 63030	0.192	0.008

## 3 Results and discussion

### 3.1 Behaviors of cavitation

The inlet flow rate could decide the intensity of the flow field inside the chamber, which would influence the development of cavitation. A series of preparatory experiments have been carried out before our experiments. We selected three groups of conditions with cavitation intensity enhanced gradually by the preparatory experiments to explore the variations of cavitation clouds. In the following discussion, three inlet flow cases including the inlet Reynolds number *Re_in, L_* = 44065 (Case I), *Re_in, L_* = 52576 (Case II) and *Re_in, L_* = 63030 (Case III) would be analyzed severally.

#### 3.1.1 *Re_in, L_* = 44065

**Fig. 3:**
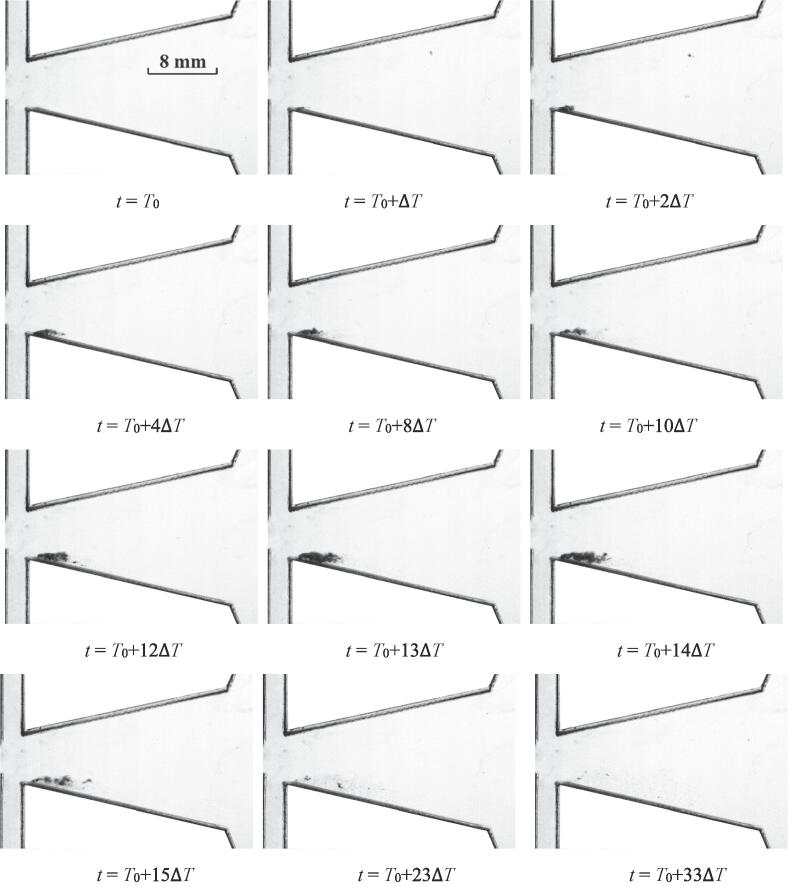
The quasi-periodical development of cavitation for *Re_in, L_* = 44065.

The development of cavitation for *Re_in, L_* = 44065 is depicted in Fig. 3. Three stages concluding the inception, expansion and collapse of the cavitation cloud could be observed. The time interval Δ*T* is 1/3600 s.

At the initial moment *t* = *T*_0_, none bubbles could be observed in the chamber. Obviously, the cavitation has not occurred at this moment. For the next moment *t* = *T*_0_ + Δ*T*, quite slight bubbles appear at the inlet wedge region. It can be implied that the cavitation inception just occurs at this moment. Soon afterwards, a little further development for the cavitation takes place. As the flow continues, the cavitation area gradually develops into a cloud-like region. At *t* = *T*_0_ + 4Δ*T*, *t* = *T*_0_ + 8Δ*T* and *t* = *T*_0_ + 11Δ*T*, it seems that three analogical cavitation clouds are moving along the inlet wedge region. Both of the location and shape of cavitation clouds for *t* = *T*_0_ + 4Δ*T* and *t* = *T*_0_ + 8Δ*T* are similar to each other. While at *t* = *T*_0_ + 11Δ*T*, there is a tendency to stretch for the cavitation cloud. In addition, it should be noted that for these three moments, quite sparse areas of cavitation bubbles could be observed at the right part of the cavitation cloud, which is named as the head of the cavitation cloud. These sparse bubbles should come from the main body of cavitation cloud. In other words, the flow velocities of the bubbles in the cavitation cloud would not necessarily maintain consistent. A fraction of bubbles located at the head part can diffuse into the water.

It seems that the cavitation inception occurs at the inlet wedge region. Then the evolution of cavitation is roughly along the wall of the chamber. At *t* = *T*_0_ + 10Δ*T*, a cavitation cloud with relatively large intensity could be observed. Whereafter, the cavitation cloud starts the process of decay at *t* = *T*_0_ + 15Δ*T*. It can be implied that the collapse of cavitation takes places in this stage.

#### 3.1.2 *Re_in, L_* = 52576

**Fig. 4:**
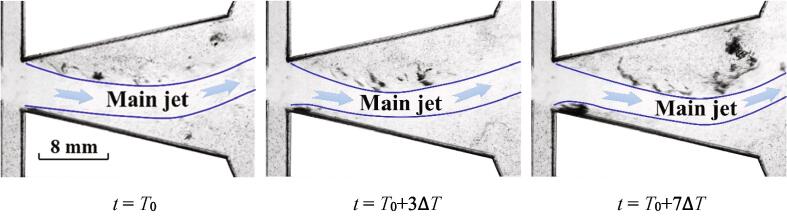
The quasi-periodical development of cavitation for *Re_in, L_* = 52576 (Stage I).

For this case, the process of the cavitation inception is depicted in Fig. 4. Since the flow rate is larger than that of *Re_in, L_* = 44065, the intensity of cavitation for this case is stronger as well. The main jet of water could be distinguished. The main jet is oscillating into the bottom wall. The low pressures between the wall and main jet [25] arouse the expansion of cavitation.

**Fig. 5:**
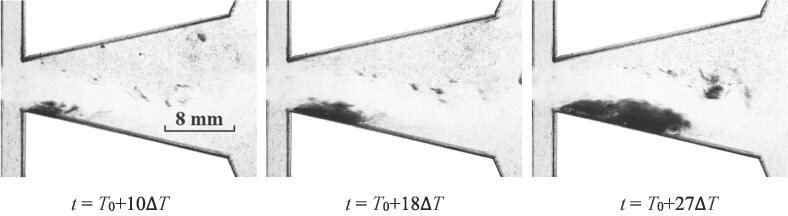
The quasi-periodical development of cavitation for *Re_in, L_* = 52576 (Stage II).

The further development of cavitation cloud is shown in Fig. 5. Quite significant expansion for the region of cavitation could be observed. This region is still located between the wall and the main jet. In this progress, the main jet is deflecting into the other hand of the chamber with the expansion of cavitation. The pressures between the main jet and the bottom wall are still in a low state. As the movement of main jet actuates the variations of the pressures, it can be noted that the development of cavitation is driven by the oscillation.

**Fig. 6:**
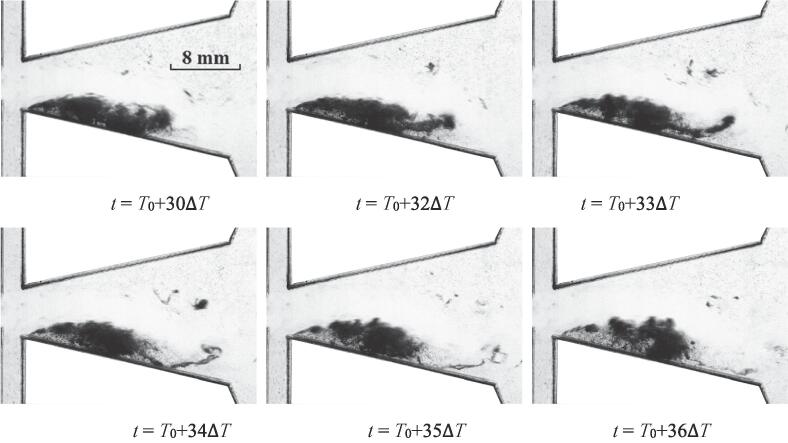
The quasi-periodical development of cavitation for *Re_in, L_* = 52576 (Stage III).

As the flow continues, it seems that the region of cavitation could maintain stable roughly for a period of time. As noted above, a fraction of bubbles located at the head part can diffuse into the water for Case I. This tendency seems more obvious for Case II as depicted in Fig. 6. At the moment *t* = *T*_0_ + 32Δ*T*, it seems that a length of vimineous cavitation bubbles would detach from the main part of the cavitation region. At *t* = *T*_0_ + 33Δ*T*, this part becomes more vimineous. It is speculated that the vortex structure located between the main jet and the nether wall may drive this process. During this period, little evolution takes place for the main part of the cavitation cloud. It should be noted that the main jet is still oscillating.

After a further period of flow, the collapse of the cavitation starts to take place. It seems that the collapse first occurs at the location circled by the blue line at *t* = *T*_0_ + 50Δ*T*, which means that the pressure of this region is in the recovery stage. While the cavitation cloud circled by the red line is still at the stage of vigorous development. A lot of vortices are moving in this area, which lead to a low pressure region and vigorous cavitation cloud. It may be implied that the oscillation of main jet promotes the pressure fluctuations of flow field in different regions, and further leads to the evolution of cavitation. Actually, the cavitation in this region does not last long. The collapse of cavitation cloud finally finishes at *t* = *T*_0_ + 88Δ*T*, which means the end of the cavitation for this period.

#### 3.1.3 *Re_in, L_* = 63030

If the flow rate of water further increases, a stable cavitation flow would develop as depicted in Fig. 8. It is difficult to distinguish the three stages concluding the inception, expansion and collapse of the cavitation cloud for this case.

### 3.2 Variation characteristics of cavitation intensity

**Fig. 7:**
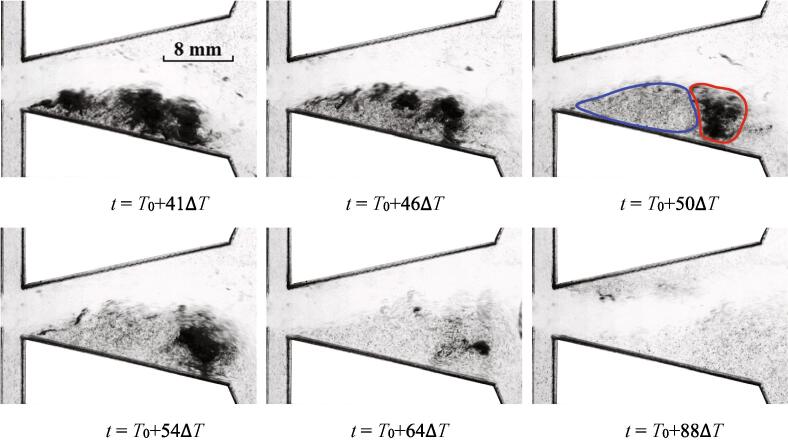
The quasi-periodical development of cavitation for *Re_in, L_* = 52576 (Stage IV).

**Fig. 8:**
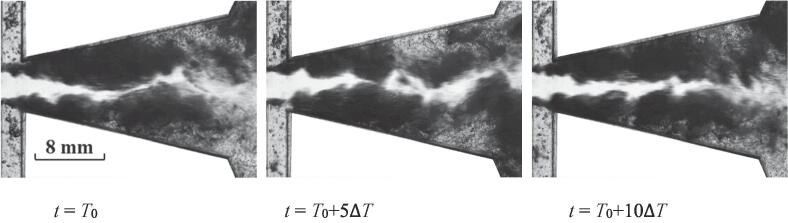
The quasi-periodical development of cavitation for *Re_in, L_* = 63030.

**Fig. 9:**
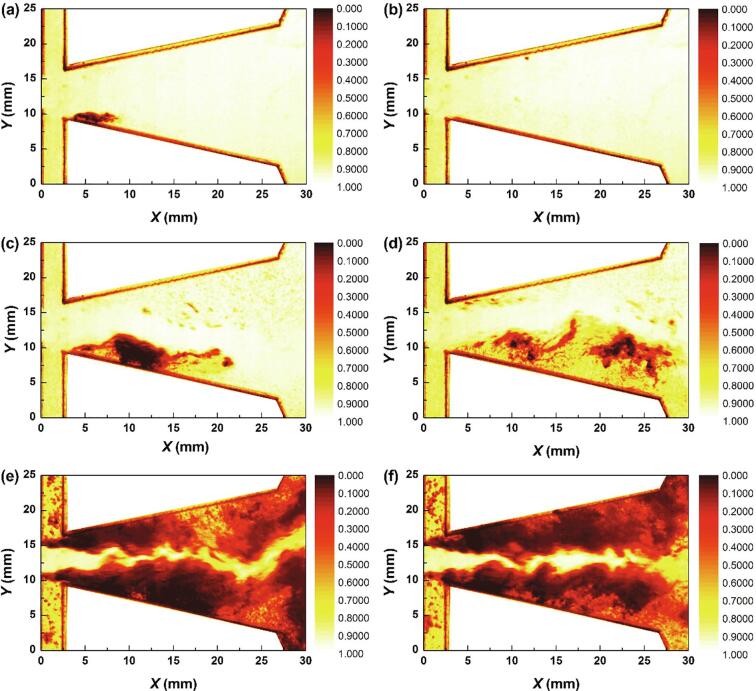
Distributions of gray intensity for different conditions.

As mentioned earlier, the grey intensity of the images is the volume of bubbles formed. Meanwhile, the cavitation intensity is proportional to the volume of bubbles formed. For one region with a relatively large grey intensity, the volume of bubbles formed is also relatively large. It means that the cavitation intensity is relatively strong. Then it can be assumed that the cavitation intensity could be represented by the gray intensity of the image, which was also proposed in the work of Liu et al. [39]. In order to further explore the development of the cavitation, the dimensionless gray intensity value $I^{*}=\left(I-I_{\min}\right)/\left(I_{\max}-I_{\min}\right)$ (*I* is the grayscale obtained by Eq. (1) while *I_max_* is the maximum grayscale and *I_min_* is the minimum grayscale in one image) which has been employed in Liu et al. [39] is introduced. As noted in Fig. 3, Fig. 4, Fig. 5, Fig. 6, Fig. 7, Fig. 8, the dark areas represent the cavitation bubbles with relatively large gray intensity values. The maximum values in the cavitation bubble areas would be much smaller than the gray intensity values in the water region. Then the smaller the dimensionless gray intensity value, the more intense the cavitation as shown in Fig. 9. It can be noted that the gray intensity could be employed to reflect the cavitation intensity. A low gray intensity means a high cavitation intensity.

**Fig. 10:**
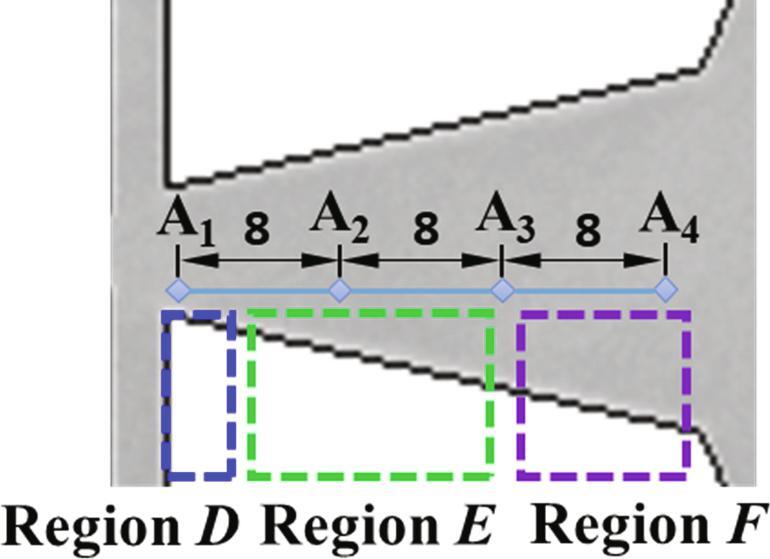
Four points along the flow direction.

**Fig. 11:**
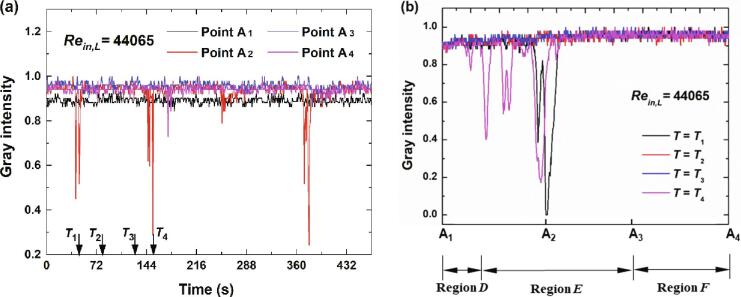
The variations of gray intensity for *Re_in,L_* = 44065.

Four points as depicted in Fig. 10 are chosen to further explore the cavitation details along the flow directions of the main jet. For a relatively low inlet flow rate (*Re_in,L_* = 44065), the variations of gray intensity at four points in Fig. 10 are depicted in Fig. 11(a). Obviously, a mutation for the cavitation intensity would occur periodically in Point A_2_. In addition, the instantaneous cavitation intensity for Point A_1_ is stronger than that of Point A_4_ while the instantaneous cavitation intensity for Point A_4_ is stronger than that of Point A_3_. It means that along the flow direction of the main jet, the cavitation intensity would decrease first and then increase.

The distributions of gray intensity along Line A_1_A_4_ at four moments are demonstrated in Fig. 11(b). The stage between *T*_1_ and *T*_4_ may be regarded as a period for the cavitation. At *T* = *T*_1_ and *T* = *T*_4_, the mutation occurs in Point A_2_. Quite minor fluctuations of the distributions for gray intensity of could be observed at *T* = *T*_2_ and *T* = *T*_3_, which means that there is almost no cavitation at these two moments. While at *T* = *T*_1_ and *T* = *T*_4_, the cavitation develops in the region that is near the entrance of the chamber. The formed cavitation cloud would collapse completely with the further flow of the main jet. For this case the whole process of cavitation would complete within Region *D* and Region *E*. A low pressure region would form during the oscillation of main jet as noted in Wu et al. [25], which induce the occurrence of cavitation.

**Fig. 12:**
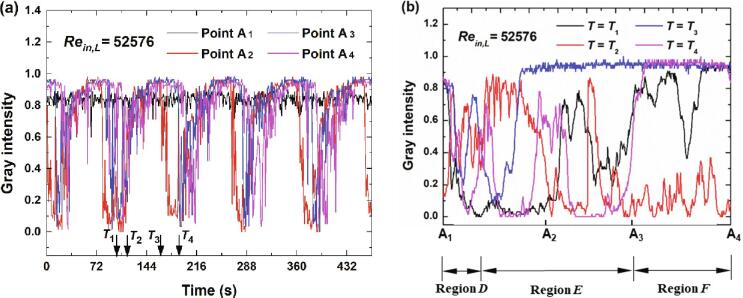
The variations of gray intensity for *Re_in,L_* = 52576.

For the case that inlet flow rate increases a little (*Re_in,L_* = 52576), the variations of gray intensity at the four points are depicted in Fig. 12(a). The average cavitation intensity for Point A_1_ still maintains the state of small fluctuations while large fluctuations could be found for Points A_2_, A_3_ and A_4_. From the variations of these three points, it could be implied that the cavitation develops along Line A_1_A_4_ roughly. The distributions of gray intensity at the four moments in Fig. 12(a) are depicted in Fig. 12(b). At *T* = *T*_1_, the cavitation mainly appear in Region *E* with certain slighter intensity of cavitation being in Region *F*. At *T* = *T*_2_, it seems that the cavitation cloud in Region *E* has collapses while vigorous cavitation cloud has moved to Region *F*. At *T* = *T*_3_, cavitation cloud exists mainly in Region *E*. From *T* = *T*_3_ to *T* = *T*_4_, the cavitation cloud gradually develop from Region *E* to Region *F*. For this case, except for the low pressure region of Region *E*, the vortexes in Region *F* are able to generate a sufficient low pressure region to induce the generation of cavitation.

**Fig. 13:**
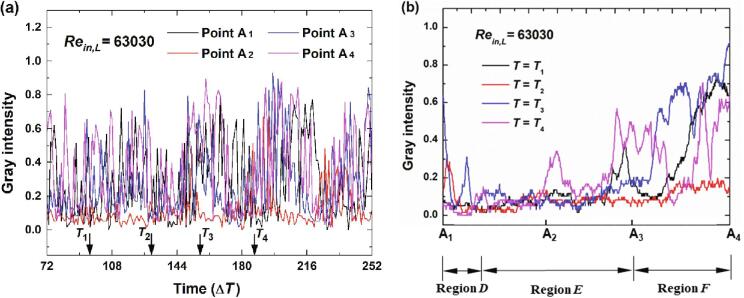
The variations of gray intensity for *Re_in,L_* = 63030.

For the relatively large inlet flow rate (*Re_in,L_* = 63030), violent fluctuations could be found for all of the four points as depicted in Fig. 13. It has been noted from the images that it is hard to distinguish the three stages for this case. While some details may be analyzed from these fluctuations. It seems that the variations of Point A_1_, Point A_3_ and Point A_4_ are similar to each other. A complete cavitation period with the three stages could be found for these three points. Nevertheless, for Point A_2_ the cavitation seems to be able to continually at the stage of vigorous development. It means that the flow fields generated by the main jet here always at the state of low pressures. The pressures at other region would keep oscillating with the movement of the main jet.

**Fig. 14:**
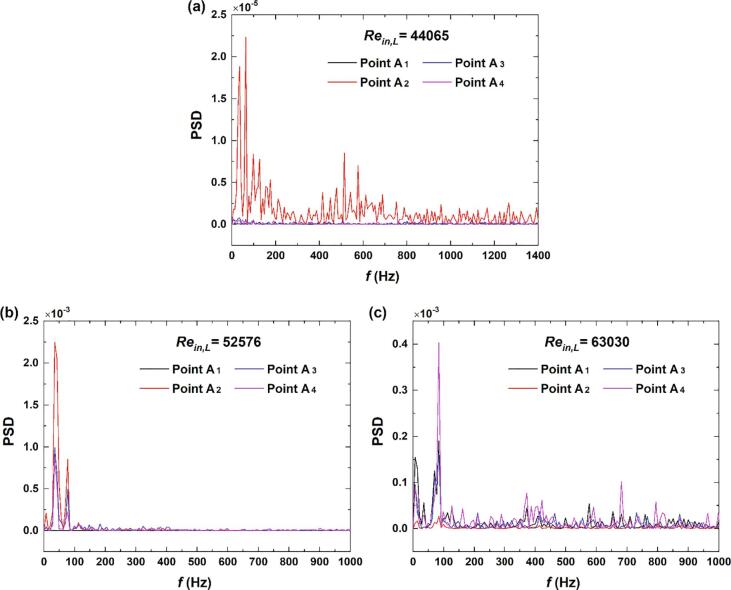
PSD of Points A_1_∼A_4_ under different inlet *Re.*

By summarizing the above three cases, it is noted that three regions could be divided as depicted in Fig. 2. In Region *D*, the main jet just flow into the chamber. The inception of cavitation would occur in this region. For relatively low inlet flow rates, little fluctuations of the gray intensity could be observed. In Region *E*, the cavitation is at the stage of vigorous development for all of the three cases. In region *F*, the collapse of cavitation takes place periodically. Nevertheless, due to the existence of low pressure vortex in this region, a certain amount of cavitation bubbles would show up in this region especially for a high inlet flow rate. In addition, the PSD of Points A1∼A4 under different inlet *Re* could be seen in Fig. 14. For low inlet liquid flow rates, the gray intensities near the inlet of the chamber are relatively larger, which could further indicates that vigorous development for cavitation occurs in this region. It is worth noting that the gray intensities of A_2_ for *Re_in,L_* = 52576 seems to surpass those of *Re_in,L_* = 63030. It means that although the cavitation for a high inlet liquid flow could be fully developed in the chamber. The cavitation intensity in the vigorous development stage would not necessarily be stronger than that of a relatively low inlet liquid flow rate.

### 3.3 Effects of cavitation number

The extent of cavitation is often described by the cavitation number σ, which is defined by [3]:

(2) $$\sigma =\frac{p_{2}-p_{v}}{\frac{1}{2}\rho U^{2}}=\frac{p_{2}-p_{v}}{p_{1}-p_{2}}$$

where $p_{v}$ and ρ are the saturated vapor pressure and density of water, respectively, and *U* is the velocity at the entry nozzle. *p*_1_ and *p*_2_ are the pressures of the inlet and outlet respectively, which are obtained by the pressure sensors as shown in Fig. 1. The experiment was carried out at an ambient temperature of 25 °C with water as the working fluid. Then the saturated vapor pressure is 3169 Pa. The values of *p*_1_, *p*_2_ and *σ* for the three cases are shown in Table 2. It should be noted that the pressures obtained from the pressure sensors are gauge pressures. The absolute pressure is the sum of gauge pressure and atmospheric pressure.

**Table 2:** The measured pressures and the obtained cavitation number.

	*p*_1_ (MPa)	*p*_2_ (MPa)	*σ*
*Re_in, L_* = 44065	0.098	0.005	1.10
*Re_in, L_* = 52576	0.144	0.005	0.74
*Re_in, L_* = 63030	0.192	0.008	0.57

It can be noted that for *Re_in, L_* = 44065 the cavitation number is larger than 1 while for *Re_in, L_* = 63030 he cavitation number is about 0.5. It has presented in the above discussion that high intensity cavitation occurs for *Re_in, L_* = 63030 yet relatively low intensity cavitation occurs for *Re_in, L_* = 44065. In the work of Shi et al. [20], a remarkable increase of the cavitation yield could be observed under the condition that cavitation number varies from 1.4 to 0.3 in a Venturi tube. It means that the cavitation in the fluidic oscillator fits the same pattern.

## 4 Conclusions

A high-speed camera experiment is conducted to reveal the characteristics of hydrodynamic cavitation generated in one self-excited fluidic oscillator. A kind of image processing method is introduced to further explore the details of cavitation. The images obtain from the high-speed camera system are employed to describe several development stages of the hydrodynamic cavitation.

(1) Hydrodynamic cavitation indeed occurs in one self-excited fluidic oscillator for certain inlet flow rates. The cavitation inception occurs at the inlet wedge region. Then the evolution of cavitation is roughly along the flow direction of the main jet.
(2) Three regions could be divided according to the distance from the entrance. For a relatively low inlet flow rate, the whole process of cavitation could complete within the region which is second nearest the entrance as a low pressure area appears periodically in this region. For a high inlet flow rate, the vortexes in the region farthest from the entrance are able to generate sufficient low pressures to induce the generation of cavitation.
(3) The intensity of cavitation could be reflected by the cavitation number in a self-excited fluidic oscillator. If the cavitation number is larger than 1, lower intensity of cavitation would occur. A violent cavitation takes place under the condition that cavitation number is less than 0.5.

### CRediT authorship contribution statement

**Gang Liu:** Funding acquisition, Investigation, Methodology, Writing – review & editing. **Haiyan Bie:** Funding acquisition, Investigation, Methodology, Writing – review & editing. **Zongrui Hao:** Funding acquisition, Methodology, Supervision. **Yue Wang:** Visualization. **Wanlong Ren:** Data curation. **Zhili Hua:** Investigation.

## Declaration of Competing Interest

The authors declare that they have no known competing financial interests or personal relationships that could have appeared to influence the work reported in this paper.
